# Eye-Neck Integrated Ultrasound in Idiopathic Intracranial Hypertension and Cerebral Venous Sinus Thrombosis

**DOI:** 10.3389/fneur.2021.696665

**Published:** 2021-07-21

**Authors:** Li Liu, Yingqi Xing, Ying Chen, Xiaorui Ji, Jiaojiao Ge, Lijuan Wang

**Affiliations:** ^1^Department of Neurology, The First Hospital of Jilin University, Changchun, China; ^2^Department of Neurology, Changchun People's Hospital, Changchun, China; ^3^Department of Vascular Ultrasonography, Xuanwu Hospital, Capital Medical University, Beijing, China

**Keywords:** idiopathic intracranial hypertension, cerebral venous sinus thrombosis, ultrasound, optic nerve sheath diameter, blood flow volume

## Abstract

**Background:** The clinical presentation of cerebral venous sinus thrombosis (CVST) overlaps with that of idiopathic intracranial hypertension (IIH), but no screening tool exists. We investigated the role of eye-neck integrated ultrasound in the diagnosis and differentiation of IIH and CVST.

**Methods:** Twenty IIH patients, 30 CVST patients, and 40 healthy controls were retrospectively analyzed. The ultrasonographic optic nerve sheath diameter (ONSD) and hemodynamic characteristics of the internal jugular veins (IJVs) were recorded. The cerebrospinal fluid opening pressure was measured after ultrasonic examination.

**Results:** The ONSD was significantly larger in IIH patients than in controls (4.71 ± 0.41 vs. 3.93 ± 0.24 mm, *p* < 0.001). The ONSD cut-off for IIH diagnosis was 4.25 mm (AUC = 0.978; 95% CI: 0.95–1.0, *p* < 0.001, sensitivity: 90%, specificity: 93%). In the CVST group, 22 (73.3%) patients had elevated intracranial pressure (ICP); the mean ONSD was significantly higher in patients with increased ICP than in those without (4.43 ± 0.33 vs. 3.95 ± 0.17 mm, *p* < 0.001). The mean blood flow volume (BFV) was significantly reduced in CVST patients (425.17 ± 349.83 mL/min) compared to that in controls (680.37 ± 233.03 mL/min, *p* < 0.001) and IIH patients (617.67 ± 282.96 mL/min, *p* = 0.008). The optimal BFV cut-off for predicting CVST was 527.28 mL/min (AUC = 0.804, 95% CI: 0.68–0.93, *p* < 0.001, sensitivity: 80%, specificity: 78%). The velocity of the unilateral IJVs-J3 segment decreased or remained constant during deep inspiration (abnormal respiratory modulate blood flow test, ARMT) in 32.5% of controls, with no bilateral ARMT. The prevalence of bilateral ARMT was 25% in IIH patients (χ^2^ = 12.9, *p* = 0.005) and 27% in CVST patients (χ^2^ = 17.6, *p* = 0.001).

**Conclusion:** Eye-neck integrated ultrasound is an easily available bedside technique to assess ICP and hemodynamic characteristics of IJVs. ONSD measurement can identify patients with increased ICP, and reduced IJV BFV may aid the differentiation of CVST and IIH.

## Introduction

Idiopathic intracranial hypertension (IIH), also known as pseudotumor cerebri, is a syndrome characterized by raised intracranial pressure (ICP) without hydrocephalus or mass lesions, normal cerebrospinal fluid (CSF) composition and normal neuroimaging results. Typical symptoms include headache, visual impairment, nausea, and papilledema (1). Despite improvements in imaging and uniformity in diagnosis, overdiagnosis of IIH reportedly occurs in 39.5% of patients (2). In a series of 131 patients presenting with papilledema and clinically suspected IIH, 9.4% of patients were eventually diagnosed with cerebral venous sinus thrombosis (CVST) (3).

CVST constitutes a serious cerebrovascular disorder characterized by a wide spectrum of symptoms and onset types. The most frequent clinical manifestations are headache, seizures, altered consciousness, and neurological focal signs on physical examination (4, 5). Diagnosis is typically based on clinical suspicion and imaging confirmation. Headache is the most common symptom and can be the only manifestation in absence of intracerebral lesion (6). As the clinical spectrum of signs and symptoms of CVST overlaps with that of IIH, but each disease has a distinct pathogenesis and treatment, the distinction between IIH and CVST is vital.

Studies show that stenosis at the junction of the transverse sinus and sigmoid sinus affects venous flow and therefore CSF dynamics in IIH (7). As a continuation of the intracranial venous sinus, the internal jugular veins (IJVs) are the main pathway for cerebral venous drainage (8). Therefore, we hypothesized that IJV's hemodynamic characteristics may be used as a parameter to prompt intracranial venous sinus thrombosis or stenosis. Ultrasonography assessments of the optic nerve sheath diameter (ONSD) are a non-invasive and dynamic method of detecting ICP changes, and may be useful for diagnostic purposes in patients with IIH (9–11). Thus, this study aimed to explore eye-neck integrated ultrasound applications in the diagnosis and differentiation of patients with IIH and CVST.

## Materials and Methods

### Patients and Controls

We conducted a retrospective study in the Department of Neurology, the First Hospital of Jilin University, between January 2017 and December 2019. The study protocol was approved by the Ethics Committee of the First Hospital of Jilin University, China, and conducted in accordance with the tenets of the Declaration of Helsinki (2019-278). We recruited hospitalized patients with the following inclusion criteria: (1) age >18 years; (2) IIH diagnosed according to the revised diagnostic criteria (1): All patients exhibited signs and symptoms of increased ICP, particularly visual disturbances related to papilledema. Lumbar puncture (LP) revealed intracranial CSF pressures >250 mmH_2_O, without cytological and biochemical abnormalities, and IIH was confirmed by angiogram or magnetic resonance venography (MRV). (3) Patients with CVST were identified with the International Statistical Classification of Diseases version 10 coding of CVST based on magnetic resonance imaging (MRI) combined with MRV and/or computed tomographic venography and/or conventional angiography. (4) Each patient underwent general medical, ophthalmologic, and neurological examinations; basic laboratory investigations; and brain MRIs. The CSF opening pressure was measured by LP, performed in patients with IIH and CVST in the left lateral decubitus (recumbent) position, with the posterior CSF opening pressure recorded with the legs fully extended. (5) The control group consisted of 40 volunteers matched for age, sex, and body mass index (BMI), recruited from among those individuals undergoing physical check-ups and without neurologic disease confirmed by computed tomography or MRI. Informed consent was obtained from both patients and controls.

### Transorbital Sonography

**Figure 1:**
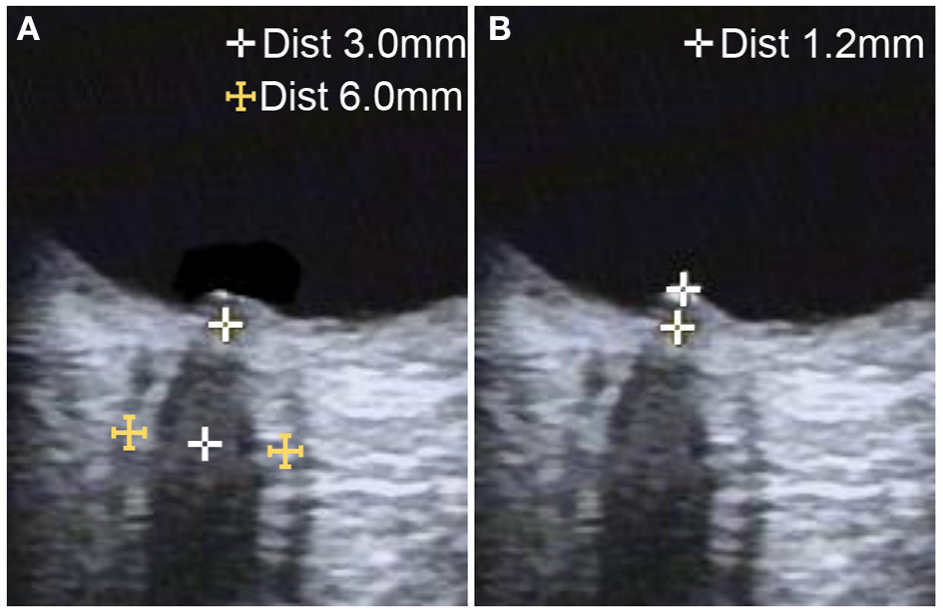
Ultrasonographic B-mode images of optic nerve measurement in a patient with idiopathic intracranial hypertension. **(A)** The optic nerve sheath diameter was measured 3 mm behind the papilla on the axial plane, showing the optic nerve in its longitudinal course. **(B)** Optic disc elevation was measured between the fundus and the dome of the papilla.

Optic nerve ultrasonography examinations were performed in B-mode using the VISION Ascendus ultrasound system (HITACHI Medical Systems, Japan) and a 5–13 MHz linear array transducer. All examinations were conducted by the same registered vascular technologist blinded to the diagnoses. The acoustic output of the ultrasonography system was adjusted according to the “as low as reasonably achievable” principle (mechanical index <0.23). Probes were placed lightly on the closed upper eyelid, which was covered with a thick gel layer to prevent pressure on the eye. According to previous protocols, the ONSD was assessed bilaterally at 3 mm posterior to the orbit, and measurements were obtained in the sagittal (with the probe in a vertical orientation) and transverse (with the probe in a horizontal orientation) planes (12–14). Mean measurements in the two scanning planes were used as the ONSD value of each eye. Papilledema was determined according to the optical disk elevation (ODE), the distance between the fundus and the dome of the papilla (Figure 1). Measurements were repeated once for each eye, and the average value was used as final result.

### Extracranial Sonography

IJVs evaluations were performed according to previous protocols (15). Participants were instructed to lie in the supine position for 10 min before scanning. Ultrasound gel was applied on the neck, with the head placed in a neutral and straight position. Neck vein compression was avoided during the examination. The time-average-mean velocity (TAMV) and IJVs' lumen were recorded immediately below the facial vein entry point (J3) (16). Manual waveform tracing over a 4 s period was used to calculate the TAMV during a brief period of apnea after normal expiration. During TAMV acquisition, the Doppler cursor was directed parallel to the vessel alignment, with the gate adjusted to comprise the entire lumen. The probe was then turned 90° at the same site of the IJVs to measure the cross-sectional area (CSA). The CSA was measured twice in B-mode and averaged for analysis. The blood flow volume (BFV) (mL/min) was defined as that equal to TAMV × CSA.

The respiratory modulate blood flow test (RMT) was also performed. If the blood flow velocity was accelerated and prolonged on deep inspiration and decreased on expiration, the RMT was defined as normal (NRMT), and if the velocity remained unchanged or decreased during deep inspiration, the RMT was defined as abnormal (ARMT). The test was performed thrice on each side to confirm the results.

IJV valvular insufficiency (IJVVI) was assessed based on previously published criteria (17). In brief, jugular valve closure was monitored during a pressure-controlled Valsalva maneuver (VM), with a forcible expiration from the patient's mouth into a flexible rubber tube connected to a manometer. Patients were asked to reach 40 mmHg of Valsalva pressure and maintain it for ≥10 s (18). A diagnosis of IJVVI was made when the duration of the reflux at the J1 level (obtained by Doppler spectrum above the venous valve) >0.88 s (19). The test was performed thrice on each side to confirm the results.

### Statistical Analysis

The required sample size was calculated as 17 participants in each group based on the receiver operating characteristic (ROC) area under the curve (AUC) under the null hypothesis of 0.5 and AUC under the alternative hypothesis of 0.8, with an α error of 0.05 and a statistical power of 91% (20). For sample size estimation, the PASS software (NCSS) was used. Statistical analyses were performed using a dedicated statistical software package (SPSS for Windows, version 23.0; SPSS Inc.). The distribution of continuous variables was assessed using the Kolmogorov-Smirnov test, and continuous variables represented as mean ± SD or median [interquartile range] (range minimum-maximum) according to their distribution. Differences between groups were examined using a *t*-test for normal distribution, and Mann-Whitney *U*-test for non-normal distribution. The correlation between the ONSD and ICP was evaluated using Pearson correlation analysis. A ROC curve was constructed to determine the optimal threshold for diagnosis. The frequencies of ARMT and IJVVI in patients with IIH and CVST and in controls were compared using the chi-squared test. The 95% confidence intervals (CIs) and two-tailed *p*-values were calculated. A *p*-value of <0.05 was considered to indicate statistical significance.

## Results

### Demographic Characteristics

The demographic characteristics of study participants are summarized in Table 1. The US examination was performed during hospital admission. The mean time from onset to US examination was 30 days in patients with IIH, and 12 days in patients with CVST.The IIH group comprised 20 patients; 16 patients with IIH underwent digital subtraction angiography, five had unilateral transverse sinus and/or sigmoid sinus stenosis, 15 bilateral transverse sinus and/or sigmoid sinus stenosis, and 12 (60%) patients received interventional therapy. The CVST group comprised 30 patients; the mean CSF pressure was ≤ 200 mmH_2_O in 8 (26.7%) patients and >200 mmH_2_O in 22 (73.3%) patients. Multiple CVST sites were found in 19 (63.3%) patients (superior sagittal sinus combined with partial or total transverse sinus and/or sigmoid sinus thrombosis, the ipsilateral IJV was involved in five patients), sigmoid and/or transverse sinus thrombosis was found in six (20.0%) patients, and sagittal or straight sinus thrombosis was found in five (16.7%) patients. Sex, age, and BMI did not significantly differ between cases and controls.

**Table 1:** Demographic characteristics of patients and controls.

	**IIH (*n* = 20)**	**CVST (*n* = 30)**	**Controls (*n* = 40)**	***p***
Age (years)	34 [11] (15–51)	34 [18] (17–72)	39 [17] (18–68)	0.110
BMI (kg/m^2^)	26 [4] (21–42)	24 [5] (14–48)	23 [5] (18–39)	0.690
Sex, Females/Males	16/4	21/9	25/15	0.183
CSF pressure (mmH_2_O)	400 [99] (250–400)	285 [58] (210–400), *n* = 8 180 [63] (110–190), *n* = 22	NA	0.001
Comorbidities	Overweight, *n* =16 Drugs (hormone replacement therapy), *n* = 2 Hepatitis, *n* = 1	Pregnancy or in puerperium, *n* = 8 Drugs (contraceptives, hormone replacement therapy), *n* = 6 Prothrombotic conditions, *n* = 5 Autoimmune disease, *n* = 2 Cancer related, *n* = 1 Hemopathy, *n* = 1		

*Values are expressed as median [interquartile range] (range: minimum-maximum); NA, not applicable; BMI, body mass index; CSF, cerebrospinal fluid; IIH, idiopathic intracranial hypertension; ICP, intracranial pressure; CVST, cerebral venous sinus thrombosis*.

### Transorbital Sonography

The mean ONSD was significantly larger in patients with IIH than in controls (4.71 ± 0.41 vs. 3.93 ± 0.24 mm, *p* < 0.001). Papilledema was present in all 30 patients with IIH, and the mean ODE was greater in this group than in controls (1.26 ± 0.25 vs. 0.65 ± 0.07 mm, *p* < 0.001). In the CVST group, 22 (73%) patients had elevated ICP (CSF pressure: >200 mmH_2_O). The mean ONSD value was 4.43 ± 0.33 and 3.95 ± 0.17 mm in patients with elevated and normal ICP, respectively (*p* < 0.001). The mean ODE value was 1.10 ± 0.33 and 0.71 ± 0.08 mm in patients with elevated and normal ICP, respectively (*p* < 0.001).

**Figure 2:**
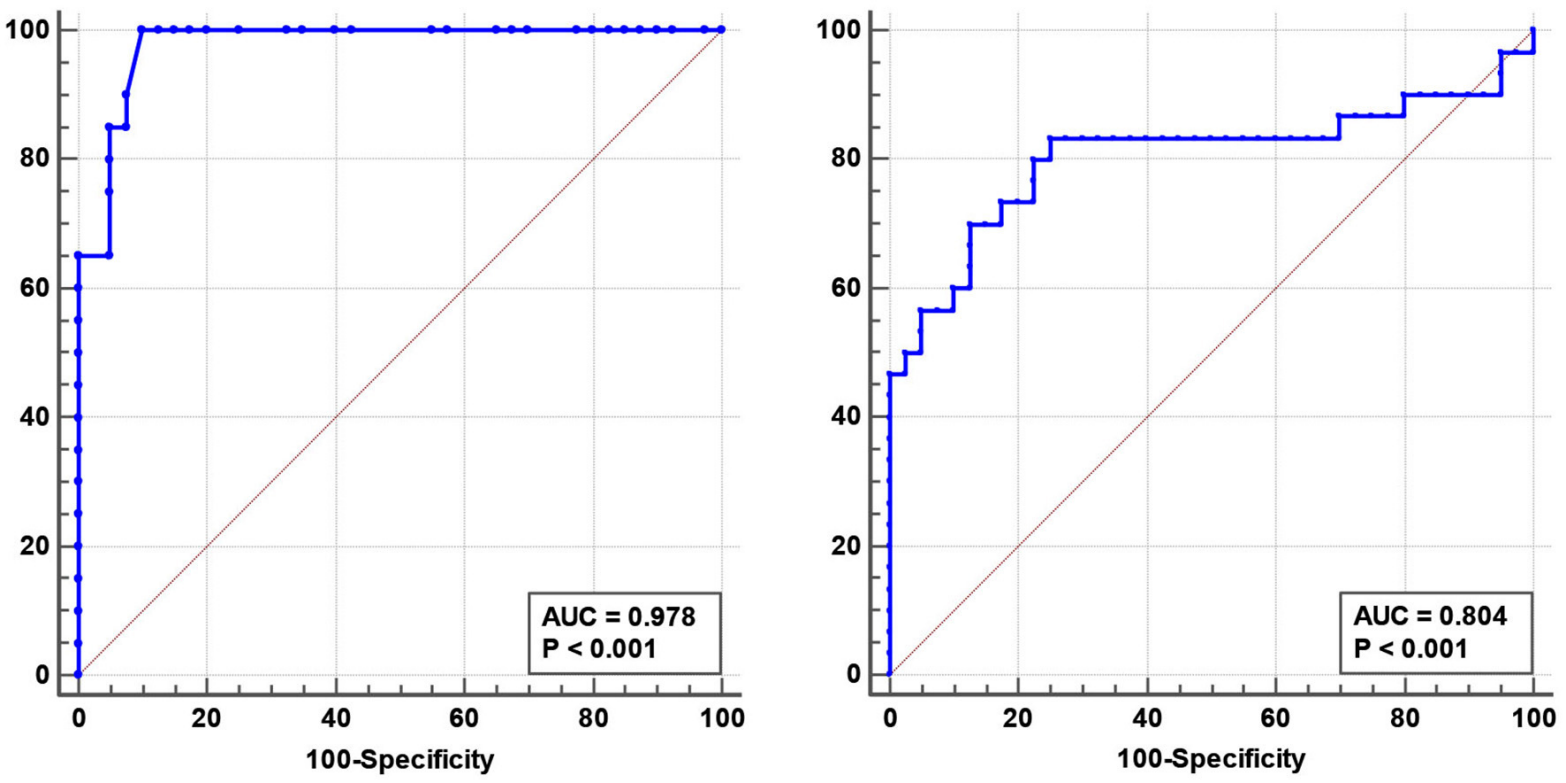
Receiver operating characteristic (ROC) curve analysis revealed that the optimal optic nerve sheath diameter predicting idiopathic intracranial hypertension was 4.25 mm (AUC = 0.978, 95% CI: 0.95–1.00 mm, *p* < 0.001) **(Left)**. The optimal blood flow volume for predicting cerebral venous sinus thrombosis was 527.28 mL/min (AUC = 0.804, 95% CI: 0.68–0.93, *p* < 0.001) **(Right)**.

There were no correlations between the ONSD and age, sex, or BMI in any group. However, the ONSD was positively correlated with the CSF opening pressure (*r* = 0.5, *p* = 0.001) in patients with IIH. ROC curve analysis revealed that the optimal ONSD cut-off value for predicting IIH (ICP >250 mmH_2_O) was 4.25 mm (AUC = 0.978; 95% CI: 0.95–1.00, *p* < 0.001, sensitivity: 90%, specificity: 93%, Figure 2).

### Extracranial Sonography

No differences in CSA, TAMV, and BFV were found between patients with IIH and controls. The BFV in the RIJV and LIJV was significantly decreased in patients with CVST compared to that in the controls (RIJV: 463.29 ± 501.84 vs. 784.11 ± 363.31 mL/min, *p* < 0.001; LIJV: 387.06 ± 360.92 vs. 576.63 ± 391.30 mL/min, *p* < 0.001) (Table 2). ROC curve analysis revealed an optimal BFV cut-off value for predicting CVST of 527.28 mL/min (AUC = 0.804, 95% CI: 0.68–0.93, *p* < 0.001, sensitivity: 80%, specificity: 78%, Figure 2).

**Table 2:** Ultrasound parameters of the flow profiles of IJVs in patients and controls.

	**Controls**	**IIH**	**CVST**	**IIH/Controls**	**CVST/Controls**	**IIH/CVST**
**LIJV-J3**						
CSA (cm^2^)	0.33 ± 0.15	0.36 ± 0.26	0.24 ± 0.11	0.527	0.008	0.062
TAMV (cm/s)	29.26 ± 14.59	29.82 ± 15.43	25.23 ± 19.93	0.892	0.354	0.198
BFV (ml/min)	576.63 ± 391.30	587.76 ± 422.23	387.06 ± 360.92	0.920	0.042	0.057
**RIJV-J3**						
CSA (cm^2^)	0.42 ± 0.18	0.34 ± 0.18	0.25 ± 0.15	0.127	<0.001	0.048
TAMV (cm/s)	38.31 ± 14.44	33.0 ± 16.23	27.0 ± 16.53	0.199	0.003	0.145
BFV (ml/min)	784.11 ± 363.31	647.58 ± 442.06	463.29 ± 501.84	0.207	0.003	0.048
mean BFV (ml/min)	680.37 ± 233.03	617.67 ± 282.96	425.17 ± 349.83	0.137	<0.001	0.008

*LIJV-J3, Left internal jugular veins-J3; RIJV-J3, Right internal jugular veins-J3; IIH, idiopathic intracranial hypertension; CVST, cerebral venous sinus thrombosis; CSA, cross-sectional area; TAMV, time-average-mean velocity; BFV, blood flow volume*.

Unilateral ARMT was observed in 32.5% of controls (20% LIJV, 12.5% RIJV), with no bilateral ARMT. However, bilateral ARMT was observed in 25% of patients with IIH (χ^2^ = 12.7, *p* = 0.005) and 27% of patients with CVST (χ^2^ = 14.9, *p* = 0.002). All cases of bilateral ARMT occurred in the patient group. IJVVI was observed in seven (17.5%) controls, five (25%) patients with IIH (*p* = 0.732), and six (20%) patients with CVST (*p* = 0.79). There was no significant difference in the incidence of IJVVI among groups.

## Discussion

In this study, we analyzed the ONSD, ODE, and hemodynamic characteristics of the IJVs in adult patients with IIH and CVST and healthy controls. Our main findings are that ultrasonography is a promising non-invasive tool for diagnosing increased ICP, and IJV BFV evaluation may further differentiate patients with CVST from those with IIH. We unexpectedly observed a decrease in unilateral venous flow, which corresponded to inspiration in some healthy controls, but the decreased bilateral IJV flow at inspiration was more likely in patients with IIH and CVST. Knowledge of the correspondence between respiration and the changes in blood flow in the IJVs may aid in the recognition of venous flow and identify the abnormalities in intracranial CSF flow.

### Transorbital Sonography

ONSD ultrasonography has been demonstrated to be a promising technique for assessing elevated ICP (12, 13). As the ONSD is distensible, the ONSD fluctuates with the CSF pressure variations (12). Our previous studies observed a linear correlation between the ONSD and elevated ICP and suggested that this method may aid in the monitoring of patients with elevated ICP (12, 13). It is also an easily learned and reproducible technique with high intra- and inter-observer reliability (14). Studies on western populations indicate that 5.0–5.9 mm is a reliable cut-off value for predicting an ICP of >200 mmH_2_O (21, 22). However, the ONSD cut-off value for the identification of an elevated opening pressure was <5.0 mm in our previous study, lower than that previously found in western populations (13). Therefore, ethnic differences should be taken into account when using the ONSD as a parameter of increased ICP.

Only recently has the ONSD been used in the diagnosis of IIH (9–11, 23). Lochner et al. performed a meta-analysis of five published studies, including a total of 96 patients with IIH (23) and found a mean ONSD of 6.2–6.76 mm. In our study, patients with IIH displayed a significantly increased ONSD, reflecting an increased ICP. Thus, papilledema could be demonstrated in all patients with IIH. The optimal ONSD cut-off value for predicting IIH was 4.25 mm (AUC = 0.978; 95% CI: 0.95–1.0 mm, *p* < 0.001, sensitivity: 90%, specificity: 93%). The cut-off obtained in the current study was significantly lower than that obtained in western populations, which underscores the need to investigate ultrasonographic ONSD of IIH patients in different ethnic groups in clinical practice.

Unlike the significant elevated ICP in patients with IIH, elevated ICP is not necessary but a frequent finding in patients with CVST (24). However, data on the utility of ONSD measurements in CVST are lacking. In our study, 73% of patients with CVST exhibited elevated ICP and an enlarged ONSD compared to patients with normal ICP (*p* < 0.001). Therefore, we propose that ONSD ultrasonography might be useful for identifying patients with intracranial hypertension and helpful in monitoring ICP in this patient population.

Papilledema is a delayed consequence of chronic CSF accumulation in the retrobulbar optic nerve sheath owing to increased CSF pressure in the cranial cavity (25). Conditions leading to increased ICP compress the optic nerve, causing stagnation of axonal transport and subsequent swelling in the optic nerve axons. Lochner et al. suggested that the ODE is not strongly correlated with ICP and may need a few days or even longer to generate (11). However, papilledema can result in insidious progressive visual loss, and an ODE finding leads to the identification of chronic ICP elevation and the importance of prompt treatment to reduce the risk of vision loss. Ultrasound can be readily performed at the bedside or in cases wherein traditional fundoscopy is difficult or impossible to assess papilledema. Our research indicated that ODE measurement appears to be indicative of optic disc swelling.

### Extracranial Sonography

In one study, 25% of patients with CVST presented with isolated headache, and another 25% presented with headache combined with papilledema or sixth nerve palsies suggesting IIH (26). As the clinical spectrum of CVST signs and symptoms overlaps with that of IIH, but each disease has a distinct pathogenesis and treatment, a distinction between IIH and CVST is vital. Unfortunately, potential pitfalls such as variation in venous anatomy, thrombus signal variability, and imaging artifacts may mimic venous sinus thrombosis, making a diagnosis of CVST insensitive (4). Therefore, an easily available method that can be performed readily at the bedside may be useful for the assessment of patients with suspected IIH or CVST.

**Figure 3:**
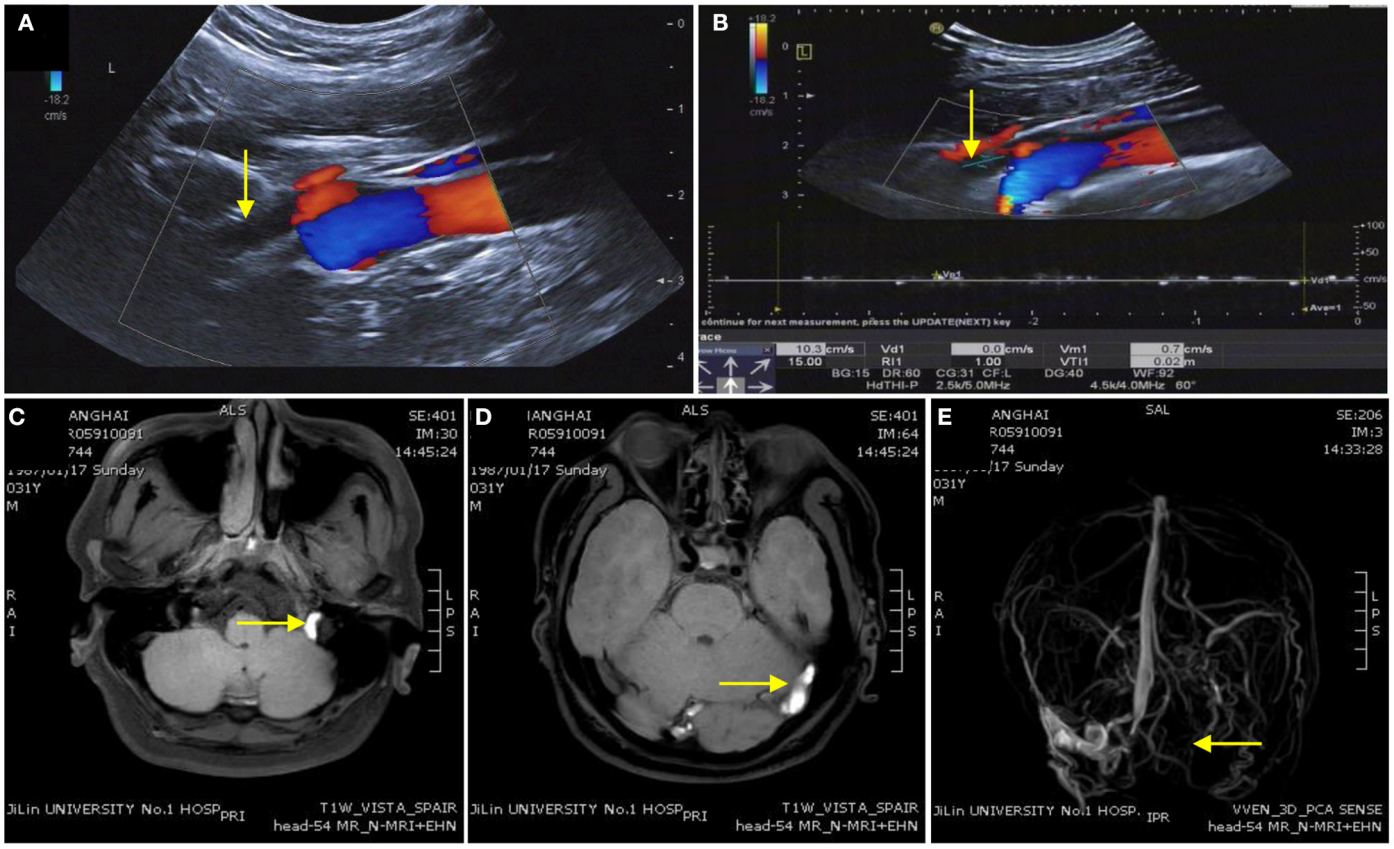
Imaging of a patient with cerebral venous sinus thrombosis. **(A,B)** Ultrasound image indicating the time-average-mean velocity of the internal jugular vein was extremely slow and unclear in the distant IJV-J3. **(C,D)** Enhanced magnetic resonance image showing the left sigmoid sinus, transverse sinus, and superior sagittal sinus thrombosis. **(E)** The superior sagittal sinus, left transverse sinus, left sigmoid sinus, and left IJV disappear on magnetic resonance venography.

Anomalous intra or extra cranial venous anatomy and consequently disturbed venous outflow and raised ICP are commonly found in IIH and CVST. Our research noted that the BFV was significantly decreased in the IJV-J3 (the distal segment of the IJVs that collects blood from the skull base) in patients with CVST (Figure 3). Ozen et al. concluded that the BFV of IJVs was significantly lower in patients with CVST than in healthy controls, and that the blood flow decreased more significantly in patients with diffuse CVST (27). Conversely, there was no statistical difference in BFV between patients with IIH and controls. These results may be related to the differences in venous sinus thrombosis and stenosis. Most patients with IIH harbor stenosis at the junction of the transverse sinus (TS) and sigmoid sinus (SS) on one or both sides (7). However, the involved lesion in patients with CVST was larger in size. In our research, 19 patients (63.3%) with CVST had multiple sites affected simultaneously, and the effect on downstream IJV hemodynamics was more pronounced in those with multiple sites affected. Ultrasound is a non-invasive, bedside, practical and frequently used technique for the imaging of neck veins that may be used for the diagnosis of CVST.

The IJVs are flexible and have a variable lumen diameter, which can be influenced by postural change, respiration, and cardiac function (28). IJVs' flow typically increases during inspiration and decreases during expiration (28). The intrathoracic pressure is negative during expiration (~-5 cmH_2_O). During deep inspiration, a more negative intrathoracic pressure (−8 cmH_2_O) would increase flow velocity and reduce IJVs' lumen (28, 29). In our research, we observed that the flow velocity decreased or remained constant in the unilateral IJV-J3 segment during inspiration in 32.5% of the controls, which seems to contradict previous studies (28, 29). When analyzing potential reasons, we first considered whether breathing-varying mechanical flow obstructions (such as muscles) could cause opposite responses to deep inspiration. In addition, asymmetry of the transverse sinuses is a common finding underlying unilateral hypoplasia or atretic sinuses in 20–39% of cases (30), which may influence blood flow regulation by respiration. However, venous sinus thrombosis or stenosis can cause variance in intracranial venous hemodynamics and increase the ICP, which may play a role in IJV dynamics. In our study, bilateral ARMT was more likely to appear in patients with IIH and CVST, which may indicate an intracranial sinus abnormality.

Nedelmann et al. suggest that patients with IIH have a higher frequency of IJVVI, which may promote venous congestion and contribute to the pathogenesis of IIH (19). In their research, fourteen out of 20 patients with IIH had either left- or right-sided, or bilaterally insufficient jugular valves, while the incidence of jugular valve insufficiency was significantly lower in the matched control group (six out of 20 individuals; *p* < 0.05). We also observed a VM-induced jugular venous reflux in patients and healthy controls and found no difference between patients with IIH, patients with CVST, and controls. Further studies are needed to include more cases and explore whether venous outflow abnormalities and obstructions underlie the development of intracranial hypertension.

Our study has several limitations. First, our observation of the respiratory modulated venous flow is only qualitative; we did not use additional sensors integrated into the ultrasound setup to synchronously measure and record the respiratory pattern with the current vascular echo graphic image. Moreover, activation of the thoracic pump should be standardized during deep inspiration. In the study by Zamboni et al., activation of the thoracic pump was standardized by setting the deep inspiration at 70% of the individual vital capacity (31). Second, double-gate MRI technique has been proposed for estimating the existence of respiratory modulation of IJV flow velocity (32), however, we choose US because of its non-invasiveness, and high-resolution images with real time dynamic information. Future studies are required to optimize imaging modalities and confirm the accuracy of these diagnostic approach. Third, angiographic and anatomical studies show wide anatomical variability and varying degrees of jugular and non-jugular venous drainage. We did not analyze other veins, such as the vertebral ones. Further research is needed to determine the hemodynamic of extracranial venous flow in case of anomalous intracranial blood vessels.

## Conclusions

Eye-neck integrated ultrasound is a fast and non-invasive technique for detecting increased ICP and evaluating IJV morphology and hemodynamic profiles during respiration. In patients with clinical features of IIH, the decrease in IJV blood flow may be useful for distinguishing CVST from IIH.

## Data Availability Statement

The original contributions presented in the study are included in the article/supplementary material, further inquiries can be directed to the corresponding author/s.

## Ethics Statement

The studies involving human participants were reviewed and approved by Ethics Committee of the First Hospital of Jilin University, China. The patients/participants provided their written informed consent to participate in this study.

## Author Contributions

LL contributed to the study conception and design, data collection, analysis and interpretation, and drafting of the manuscript. LW, YX, and YC contributed to the study conception and design, analysis and interpretation of the data, and revision of the manuscript. XJ and JG contributed to the data collection and revision of the manuscript. All authors contributed to the article and approved the submitted version.

## Conflict of Interest

The authors declare that the research was conducted in the absence of any commercial or financial relationships that could be construed as a potential conflict of interest.
